# Tumour Heterogeneity: The Key Advantages of Single-Cell Analysis

**DOI:** 10.3390/ijms17122142

**Published:** 2016-12-20

**Authors:** Marta Tellez-Gabriel, Benjamin Ory, Francois Lamoureux, Marie-Francoise Heymann, Dominique Heymann

**Affiliations:** 1 Laboratorio Hematologia Oncologica y de Transplantes, Institut Investigacions Biomèdiques (IBB) Sant Pau, Hospital de la Santa Creu i Sant Pau, 08025 Barcelona, Spain; 2 Institut National de la Santé et de la Recherche Médicale (INSERM), Unit 957, Pathophysiology of Bone Resorption and Therapy of Primary Bone Tumours, Equipe Ligue 2012, Faculty of Medicine, University of Nantes, Nantes 44035, France; benjamin.ory@univ-nantes.fr (B.O.); francois.lamoureux@univ-nantes.fr (F.L.); m.heymann@sheffield.ac.uk (M.-F.H.); 3 Department of Oncology and Metabolism, Medical School, University of Sheffield, Beech Hill Road, Sheffield S10 2RX, UK; 4 European Associated Laboratory, INSERM, Sarcoma Research Unit, Medical School, University of Sheffield, Sheffield S10 2RX, UK

**Keywords:** heterogeneity, circulating tumour cells, single cells

## Abstract

Tumour heterogeneity refers to the fact that different tumour cells can show distinct morphological and phenotypic profiles, including cellular morphology, gene expression, metabolism, motility, proliferation and metastatic potential. This phenomenon occurs both between tumours (inter-tumour heterogeneity) and within tumours (intra-tumour heterogeneity), and it is caused by genetic and non-genetic factors. The heterogeneity of cancer cells introduces significant challenges in using molecular prognostic markers as well as for classifying patients that might benefit from specific therapies. Thus, research efforts for characterizing heterogeneity would be useful for a better understanding of the causes and progression of disease. It has been suggested that the study of heterogeneity within Circulating Tumour Cells (CTCs) could also reflect the full spectrum of mutations of the disease more accurately than a single biopsy of a primary or metastatic tumour. In previous years, many high throughput methodologies have raised for the study of heterogeneity at different levels (i.e., RNA, DNA, protein and epigenetic events). The aim of the current review is to stress clinical implications of tumour heterogeneity, as well as current available methodologies for their study, paying specific attention to those able to assess heterogeneity at the single cell level.

## 1. Introduction

In oncology, molecular, cellular and architectural variability are frequently referred to with the term “heterogeneity”, a concept that increases the complexity of the pathogenesis of malignant tumours. In terms of cell phenotype, cell density or cell location, cell heterogeneity can be observed between tumours that occur in the same organ and/or between patients. Inter-tumour heterogeneity leads to the classification of tumour subtypes, which can be distinguished by whether or not their molecular profile correlate with their morphologies and expression of specific markers. In addition, tissue variation also occurs within individual tumours (intra-tumour heterogeneity), meaning that tumour cells can have various functional properties and can express several markers [1,2]. Such heterogeneity is likely to result in tumours adapting to changes in microenvironmental conditions and/or a tool for changing their malignant potential. This in turn will lead to cellular clones with different sets of undetermined hallmarks [1,3]. Tumour heterogeneity has several key clinical impacts: (i) it has been associated with acquired drug resistance; and (ii) it limits the precision of histological diagnoses and consequently reduces the value of a biopsy.

Ideally, tumour heterogeneity should be monitored over time, and more specifically in relation to therapeutic strategies. However, the invasiveness of biopsies makes it impractical to perform them frequently. The risk of cell dissemination, the procedures cost, as well as sometimes the existence of multiple metastases and the time needed must also be taken into consideration. Circulating tumour cells (CTCs) are a potential surrogate for tissue-based cancer diagnostic and may thus provide the opportunity for monitoring serial changes in tumour biology. Recent progress has made possible accurate and reliable quantification and molecular characterization of CTCs [4,5]. The present review describes different types of heterogeneity, their clinical implications, and techniques currently used to analyse them.

## 2. Types of Heterogeneity

### 2.1. Intra-Tumour Heterogeneity

Intra-tumour heterogeneity was demonstrated by Fidler and Hart more than 30 years ago in murine models [6]. It refers to the existence of distinct subpopulations of cancer cells within tumours, within various metastatic sites, and between metastatic sites and primary foci [7]. Furthermore, intra-tumour heterogeneity applies not only to tumour cells, but also to the components of their microenvironment [8]. The cancer cell populations detected differ in terms of tumorigenicity, activation of signalling pathways, evasion from antitumour immunity, induction of senescence, production of secreted factors, migration, metastasis, angiogenic capacity, genetic make-up, response to anticancer agents and activation of metabolic pathways [1,9,10]. Intra-tumour diversity is thought to develop due to either genetic (epigenetic) disorders in tumour cells themselves, or under the influence of the tumour microenvironment, or in the background of interactions between these factors [11].

**Figure 1:**
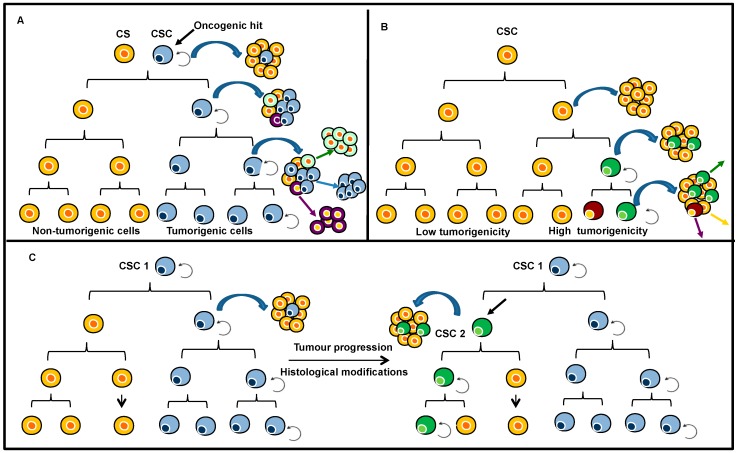
Two models for tumour heterogeneity and propagation: (**A**) In the cancer stem cell (CSC) model, only the CSCs can generate a tumour based on their self-renewal properties and enormous proliferative potential. The tumour heterogeneity is associated with the capacity of differentiation of these CSCs and series of mutations and/or epigenetic events. The other cancer cells (CS) are non tumorigenic in immunodeficient mice for instance; (**B**) In the clonal evolution model, all undifferentiated cells (CSC) have initially similar tumorigenic capacity. However, CSCs acquire a series of mutations resulting in dominant clones; and (**C**) Both tumour maintenance models may underlie tumorigenesis. Initially, tumour growth is driven by a specific CSC (CSC1). With tumour progression, another distinct CSC (CSC2) may arise as a result of clonal evolution in CSC1. This may be a result of the acquisition of an additional mutation or epigenetic modification. CSC2 is more aggressive and becomes dominant, driving tumour formation. (Adapted from Visvader, J.E.; Lindeman, G.J. *Nat. Rev. Cancer* **2008**, *8*, 755–768. Copyright 2008 Nature Reviews Cancer.).

Intra-tumour heterogeneity was initially explained by means of the cancer stem-like cell (CSC) or clonal-evolution models [12] shown in Figure 1. The CSC model is a hierarchical model in which only CSCs can generate a tumour, based on their self-renewal properties and high proliferative potential (Figure 1A). In the clonal evolution model, all undifferentiated clonal cells have similar tumorigenic ability (Figure 1B). These paradigms for tumour propagation exist in human cancer, and the two models are not mutually exclusive. However, only the CSC model is hierarchical (Figure 1C). In 1976, Peter. C Nowell described a model for cancer development combining the two previous models [13]. The result is a mutant cell that initiates a monoclonal disease. This cell exhibited higher chromosomal instability, leading to the emergence of both new clones and the polyclonal disease associated with secondary genetic events, strengthening the heterogeneity of the tumour. Mutant tumour cells with a growth advantage are then selected and expanded, and the cells in the dominant population have a similar potential for regenerating tumour growth [13]. Nevertheless, intra-tumour heterogeneity cannot be limited solely to genetic events. Numerous studies carried out on cell lines considered as having a high degree of genetic homogeneity, have shown that drug responses are also strongly associated with intercellular epigenetic heterogeneity [14]. Epigenetic mechanisms are defined by numerous processes, including DNA methylation, post-translational modification of histones, and chromatin remodelling. All of them are essential for genome organisation, gene expression and cell function [15]. The failure of cancer therapies to achieve sustainable therapeutic responses is often attributed to intra-tumour heterogeneity.

### 2.2. Inter-Tumour Heterogeneity

**Figure 2:**
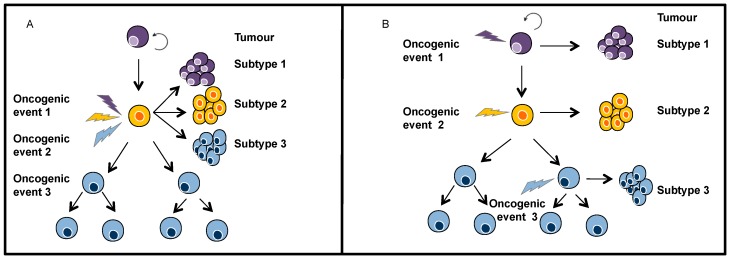
Two models of inter-tumour heterogeneity: (**A**) In the genetic and/or epigenetic mutation model, mutations/modifications primarily determine the phenotype of the tumour. For this reason, different mutations/modifications result in different tumour subtypes; and (**B**) In the cell-of-origin model, different cell populations in the lineage hierarchy are used as the cells of origin for the different cancer subtypes.

Inter-tumour heterogeneity defines differences between tumours of the same origin in different patients. These tumour subtypes have specific individual molecular signatures, different biological behaviours and, as a result, have a differential impact on clinical outcomes [16]. Two main mechanisms have been conceptualised to explain inter-tumour heterogeneity: (i) genetic mutations or/and epigenetic modifications occurring within th e same target cell and resulting in different tumour phenotypes (Figure 2A); and (ii) different tumour subtypes arising from distinct cells within the tissue that serve as the cell of origin (Figure 2B). In addition, extrinsic mechanisms may generate tumour variability, such as stromal heterogeneity (i.e., the existence of different populations of cancer-associated fibroblasts), the complexity of immune system infiltration into the tumour bulk, or dysregulation of the extracellular matrix [17]. All these mechanisms are crucial for determining malignant growth [18].

## 3. Sources of Heterogeneity

**Figure 3:**
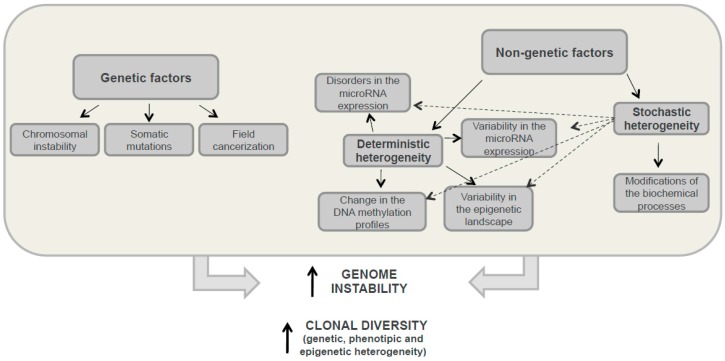
Development factors for tumour heterogeneity. This diagram shows the genetic and non-genetic mechanisms that occur in tumour cells enhancing genome instability and leading to both increased clonal diversity, and the development of genetic, phenotypic and epigenetic heterogeneity. Solid arrows indicate strict regularities and dotted arrows indicate possible relations.

Early findings suggested that genetic heterogeneity was a result of treatment pressure [19]. However, it has since been proved that genetic differences in cells within the tumour, between different metastases diagnosed simultaneously, and even within the same excised primary or metastatic tumour, can occur in the absence of any intervening treatment [20,21,22]. Phenotypic differences among cells within a tumour may reflect the genetic differences between them. However, tumour cell diversification is not only due to genetic factors, but also to different non-genetic causes such as epigenetic processes, the microenvironment or stochastic mechanisms, all of which can result in this cell heterogeneity. In the following paragraphs, we will summarize the different causes of tumour heterogeneity (Figure 3).

### 3.1. Genetic Heterogeneity

Nowell proposed that most neoplasms have their origin in a single cell, and tumour progression results from acquired genetic variability within the original clone making possible sequential selection of more aggressive subpopulations [13]. Nowadays, it is well-known that tumours consist of multiple different clones and that there are multiple genetic diversification processes for tumour development [23]. Importantly, two very recent studies of prostate-cancer metastases have revealed existence of the cross-seeding concept meaning that secondary sites can be seeded by multiple cell populations derived not only from the primary tumour, but also from other metastases [24,25].

Chromosomal instability is one of processes that generate variability. Cells capable of surviving with this genetic instability generate a mutational load and genetic diversity. Increased genetic instability is considered to be one of the cancer hallmarks [26,27]. The number of cell divisions correlates with random mutagenesis events, and thus with genomic changes. In this context, more the number of cell divisions increases, more the risk of “genetic” abnormalities (i.e., duplications, deletions, mutations and spontaneous errors of DNA replication) increases. Consequently, larger tumour size is usually associated with higher phenotypic variability. In addition, somatic mutations contribute to tumour heterogeneity as they generate different subclones that are subjected to a selection process for cancer formation [23]. The field cancerization phenomenon is defined as a transformation process that allows genetically changed but histologically normal cells to precede the development of a tumour [28], and it has been described as one of intra-tumour heterogeneity mechanisms [29]. Although genetic heterogeneity is not likely to be the primary contributor to intra-tumour phenotypic heterogeneity, in general, genetic changes are heritable and therefore some of them are expressed during tumour progression.

### 3.2. Nongenetic Heterogeneity

Nongenetic factors are associated with development of so-called deterministic and stochastic heterogeneities. Stochastic heterogeneity corresponds to differences between genetically identical cells caused by spontaneous modifications to biochemical processes in the cell when deterministic diversity develops (Figure 3). These processes can lead to transient drug resistance and can also promote the transition from one deterministic status to another [30]. The formation of intra-tumour deterministic heterogeneity can be caused by changes in the DNA methylation profile [31], epigenetic landscape variability [32], chromatin reorganisation variability in gene expression and microRNAs [33], etc. (Figure 3). In cancer, complex internal and external factors induce changes in tumour cells affecting multiple aspects of cellular biology and differentiation status [34]. Internal factors include epigenetic modifications, genetic background and degree of differentiation. MicroRNAs which regulate gene expression at the transcriptional level by binding to gene promoters, are now believed to play the most important role in generating cellular diversity within a single tumour [35]. Phenotypical changes induced by epigenetics are reversible, which makes it more difficult to study the tumour heterogeneity developed by this process.

The tumour microenvironment, including inflammatory cells, hypoxia conditions or the extracellular matrix, is the main external factor that promotes cell diversity by selecting cells adapted to various conditions in the microenvironment [30]. The most obvious example is variation in the distances from individual cancer cells to the vasculature, which leads to differential trophic supply and metabolic status of the cancer cells [18]. In addition, other stromal cells, including fibroblasts, inflammatory cells and pluripotent mesenchymal cells also contribute to the diversified genotypes and phenotypes of cancer cells by secreting cytokines, growth factors and extracellular matrix (ECM) components [19].

## 4. Heterogeneity of Distant Metastases: Circulating Tumour Cells

Differences in DNA mutational status and RNA or protein expression profiles between the primary tumour and metastases, or within a single tumour, has been widely reported [36,37,38,39,40,41,42]. This spatial heterogeneity, i.e., the coexistence of various tumour cell clones, with different characteristics, in the same or distant organs can result in suboptimal treatment. Circulating tumour cells (CTCs) constitute a heterogeneous population of tumour-derived cells that could be the precursors of metastases or/and could contribute to the primary tumour-to-metastasis or/and metastasis-to-metastasis spread. CTCs originated from the primary tumour or metastatic foci could invade the surrounding tissue, enter either the lymphatics or the bloodstream, survive in the circulation, extravasate into a tissue and finally grow at the new site [43]. Several studies support the idea that cellular heterogeneity within CTCs comprehensively reflects the full spectrum of mutations in the primary tumour and metastatic lesions better than a single primary tumour or metastatic biopsy [44,45,46].

Different studies have shown that CTCs exhibit considerable cell-to-cell diversity [44,47,48]. It has also been shown that CTC profiles evolve as the disease progresses [49,50,51]. Conventional molecular assays only reflect the signal from the dominant clone or an average signal from all the clones, even though this may not be the most malignant CTC clone. For this reason, single cell analysis may be a solution to this problem. As CTCs are present at extremely low concentrations in a background of peripheral leukocytes, molecular characterisation of CTCs in the blood remains challenging. There are currently many techniques based on a range of parameters that make it possible to enrich CTCs and single CTCs for further characterisation. These techniques include the CellSearch^®^ system (Veridex), which is the only assay for enriching and enumerating CTCs in clinics that has been cleared by the US Food and Drug Administration, or the DEParray™ technology (Silicon Biosystems, Bologna, Italy), the only automated instrument that can identify, quantify and recover individual cells after initial pre-enrichment step [5,52]. We previously described in detail and discussed advantages and pitfalls of various methodologies suitable for CTC enrichment and isolation [5].

Recent findings have identified the importance of CTC clusters in tumour dissemination. Contrary to common belief, Au et al. demonstrated that grouped CTCs are able to pass through the narrow vessels to reach distant organs [53]. This may be a major source of heterogeneity at the metastatic point, and may imply more effort for developing methodologies to isolate and characterise CTC clusters.

## 5. Clinical Implications of Tumour Heterogeneity

**Figure 4:**
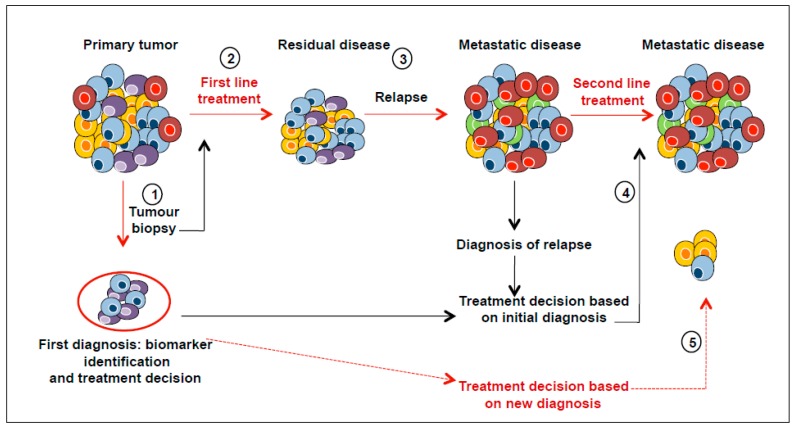
Effects of tumour heterogeneity on the predictive value of biomarkers. Cancer diagnosis is commonly based on a biopsy (**1**) that contains only a small fraction of tumour and may thus not be representative of all the subclones (cells in different colours). The first line treatment can be successful in eliminating dominant clones (**2**), but resistant clones are selected and drive disease progression (**3**). Metastases can develop from primary tumour cells, or from clones that survive the initial therapy. Therefore, the clonal composition of metastatic lesions may be completely different from that of the primary tumour sample, and treatments based on the initial diagnostic sample may be suboptimal for the treatment of metastatic disease (**4**). New diagnosis after a relapse must be made before applying a second line treatment (dashed-red lines) (**5**). (Adapted from Almendro et al.) [68].

The main reason why cancer treatments fail is that treatment programmes are not designed to address tumour heterogeneity. Most of the methods used to characterise tumours lead to a global overview of the characteristics of the cancer tissue, corresponding to an average picture of all the tumour clones and their microenvironment. The intra- and inter-tumour variations of biomarkers may affect the success of biomarker-driven clinical trials if the biomarker used to both predict the therapeutic response and stratify the patients into subgroups shows spatial variability. In addition, divergent evolution of metastatic tumour cells and different microenvironments at the metastatic sites may contribute to the change in the expression of biomarkers initially identified in the primary tumour [18,54]. Treating metastatic disease based on the biomarkers expressed in the primary tumour may thus be less than optimal. Deciding which mutations are clinically relevant, and developing an easy-to-interpret framework for reporting the results to clinicians, are complex tasks. Better understanding of tumour content and monitoring changes in cell populations during disease progression and treatment will improve both cancer diagnosis and therapeutic design (Figure 4).

It is possible that new targeted therapies introduced into clinical practice will induce temporary benefits until the tumour becomes resistant. Monitoring accumulated genetic or plastic changes in cancer during patient’s treatment might guide selection of the next therapy, or even the addition of a subsequent therapy. Ideally, researchers would obtain multiple regions from each tumour or metastasis in order to capture spatial heterogeneity. Epigenetic and other analyses also need to be performed. Clinical annotation of the samples and phenotypic correlation are essential at each step. At present, medical protocols envisage obtaining biopsies to establish diagnosis and determine whether the predictive biomarkers are in agreement or instead present differences between the metastases and primary tumours. However, most metastases are difficult to access, and biopsies are invasive, inconvenient and costly. Obtaining subsequent biopsies is thus almost impossible from a logistic point of view. To overcome these problems, detecting and characterising CTCs might be an excellent alternative.

Studies on CTCs have focused on their prognostic significance, their utility in real-time therapies monitoring, the identification of therapeutic targets, resistance mechanisms and understanding the metastatic process [55,56]. Recently, it has been shown that molecular characterisation of CTCs is pivotal for increasing the diagnostic specificity of CTCs and their potential as therapeutic targets [57]. As shown in several studies, CTCs are heterogeneous [58,59,60,61,62]. Single-cell analysis of CTCs has thus been established as the most reliable method. Dynamic changes or transitions in the biomarker status of CTCs may reflect the presence of the selective pressure that can be exerted by therapeutic interventions. Performing serial measurements of the dynamics of longitudinal biomarkers displayed in CTCs over the course of multiple sequential therapies, may provide insight into tumour evolution [63]. Furthermore, monitoring temporal CTC heterogeneity may help to identify the most effective drugs in individual cancer patients [64,65]; especially those who already have or will soon have tumours that are resistant to anti-cancer treatments. As an example, one of the most studied markers is PIK3CA. It has been shown that somatic mutations in PIK3CA play a crucial role in therapy response in breast cancer. Monitoring the mutational status of PIK3CA marker may thus improve the success of treatment decisions in these patients [66]. Finally, an interesting application for the study of tumour heterogeneity in clinical practice is related to predictive biomarkers of tumour evolution. Existence of spatial and temporal heterogeneity in the expression of diagnostic markers, between cells in the same tumour region or at the same stage of the disease, limits success of current predictive biomarkers. Ideally, identifying drivers or suppressors of genome instability in solid tumours, whose activation or inactivation is required to initiate intra-tumour heterogeneity and diversity, could provide a tractable means of ultimately attempting to limit tumours evolutionary processes [3]. As an example, a study by Kovacs et al. revealed the existence of large-scale genome instability signatures that are characteristic of BRCA1/2-deficient tumours in more than 80% of analysed osteosarcoma samples [67].

## 6. Methods for Studying Tumour Heterogeneity

In the following section, we will review available methodologies for studying tumour heterogeneity in tissues and at the single cell level. Table 1 summarizes the main characteristics of these methods.

### 6.1. Cell heterogeneity in Tissues

Assessing heterogeneity in tissue samples has been limited to methods based on microscopy, such as immunohistochemistry (IHC), immunofluorescence (IF) and fluorescence in situ hybridisation (FISH). In situ hybridisation techniques make it possible to detect RNA, DNA and protein molecules with high sensitivity in both frozen and formalin-fixed paraffin-embedded (FFPE) tissues [69,70]. These techniques are normally the preferred methods for studying tumour heterogeneity for several reasons: there are many available samples as they are a routine part of pathological diagnostic; they preserve the specificity of tissue context and they have been used to investigate tumour traits at the single-cell level. However, these methods suffer from difficulties in terms of quantifying expression levels and comparing results within and between samples, owing to variable backgrounds and target accessibility. In addition, the main limitation is the use of these techniques for high throughput analysis.

Immuno-FISH identifies genomic imbalances and specific DNA translocations, in particular cell subpopulations by combining the hybridisation of probes with the immunodetection of antigenic markers [71]. Comparative Genomic Hybridisation array (a-CGH) can be used to map genome-wide copy number variations in FFPE tissues. This technology can also be used to analyse single cells [72,73]. The RNAscope technique (Advanced CellDiagnostics Inc., Hayward, CA, USA) makes it possible to investigate multiple transcripts in the same tissue section at high-resolution [74]. New types of mRNA probes have significantly improved analyses and quantification of spatial gene-expression patterns at the single-cell resolution level; this information has been used to identify cells expressing particular mRNAs in specific anatomic areas [69]. Recently, Lee et al. described new technology for gene expression profiling intact cells and tissues called Fluorescent in situ sequencing (FISSEQ) of RNA [75]. Another very recent method is STAR-FISH, a technique based on combining in situ PCR and FISH to make possible the simultaneous detection of point mutations and copy number variations at the single-cell level in high quality FFPE samples. Limitations of STAR-FISH include the need for a priori knowledge of the mutation to be analysed and the number of fluorochromes that can be applied to detect in situ PCR products and chromosomal regions [76].

Heterogeneity at the protein level has mainly been investigated by targeted assays, using antibodies for IHC, or after extraction by reverse phase protein array or liquid chromatography. Matrix-assisted laser desorption/ionisation (MALDI) and multiplexed ion beam imaging are performed in situ while preserving the morphological characteristics [77]. Evaluating multiple markers at the single-cell level is easier to perform using immunofluorescent antibodies which are useful for identifying specific signalling pathways in defined cell subpopulations and/or for identifying the topological locations of cell subpopulations within a tumour [68]. Very recently, Sood et al. published the developmennt of a multiplexed immunofluorescence staining platform to measure the expression of 27 proteins at the single-cell level in formalin-fixed and paraffin-embedded samples [94].

### 6.2. Cell Heterogeneity at the Single-Cell Level

As mentioned in the previous sections, single-cell analysis is the most reliable approach for studying tumour heterogeneity. This section briefly summarises the technologies most used today to identify molecular differences at the single-cell level. Advances in Whole Genome Analysis (WGA), Next Generation Sequence (NGS), fluorescence-activated cell sorting (FACS) and other techniques have made it possible to analyse multiple markers in single tumour cells isolated from fresh or fixed primary tumours and metastases [78,79,95,96], and attempts have also been made to use these techniques to explore tumour heterogeneity in individual CTCs [44,61,80,81,82], isolated from blood or bone marrow.

#### 6.2.1. Single-Cell Genomic and Transcriptomic Analyses

Single-cell technologies have evolved considerably in the area of genome and transcriptome sequencing in the last ten years. The first challenge is obtaining a suspension of viable single cells from complex solid tissues by means of mechanical or enzymatic dissociation, or by using more sophisticated technologies such as laser-assisted microdissection [83]. Once in suspension, single cells must be isolated by serial dilution, micropipetting, or FACS, among others [84]. CTCs from whole blood or bone marrow are enriched using methods specifically developed for this purpose and reviewed elsewhere [5,97]. To study DNA heterogeneity, the next step is to perform WGA in order to amplify the single-cell genomes and minimise the introduction of artefacts. There are three main WGA methods: (i) pure PCR-based amplification (DOP-PCR); (ii) isothermal amplification (MDA); and (iii) hybrid methods (MALBAC or PicoPLEX), which are the most commonly used. Performing single-cell MDA in microfluidic emulsions seems to markedly improve the uniformity of amplification and decrease contamination. Many groups have had success with this approach [98].

It is thus necessary to define the type of genomic interrogation of the amplified genomes. Depending on the objective of the study, it is possible to query specific loci of interest (either by target-specific amplification using PCR, or target capture through hybridisation) [98]; sequence all the exome [95]; or sequence the entire genome, called Whole Genome Sequencing (WGS) [78]. This last method provides more information than the other two approaches, such as the single nucleotide variant (SNVs), copy number variants (CNVs) and non-coding and structural variants. The counterpart is the high cost. Determining genetic heterogeneity between single cells after WGS is currently achieved by applying model-based clustering, which makes it possible to include false-negative errors [99].

If the aim of the study is to explore transcriptome heterogeneity (Whole Transcriptome Sequencing (WTS)), single-cell RNA sequencing methods must be applied. Nowadays, there are many protocols available for RNA sequencing at the single-cell level, Quartz-seq, CEL-seq, STRT-seq, Smart-seq or Drop-Seq are the best known, each one having both advantages and disadvantages [85,86]. A very recent method described by Chen et al. is multiplexed error robust FISH (MERFISH), a single-molecule imaging method that allows thousands of RNA species to be imaged in single cells by using combinatorial FISH labelling with encoding schemes capable of detecting and/or correcting errors [87].

Regardless of which single cell analysis is performed, WGS or WTS, and given high number of technical errors that emerge during the process, the results must be validated, as these technical errors are often interpreted as real biological variations [100]. Despite initial restrictions of the “omics” techniques in the field of CTC research, many studies have now been described using these technologies to investigate heterogeneity in single CTCs. These studies include that by De Luca et al., in which inter- and intra-patient heterogeneity is observed in the mutational status of breast cancer CTCs analysed by NGS [101]. In addition, there have been studies comparing CTCs and metastases or primary tumours at the single-cell level, such as the study performed by Jiang et al. in which they discovered highly heterogeneous short structural variants of PTEN, RB1, and BRCA2 in all tumour and CTC prostate samples assessed using high-quality WGS [102]. Interestingly, Powell et al. performed one of the first studies in CTCs focused on single-cell transcriptomes. They used a microfluidic platform (Fluidigm) to perform multiplexed quantitative PCR (qPCR) on 87 cancer genes in different breast cancer samples. Importantly, they found high transcript levels of genes associated with metastases: NPTN, S100A4, S100A9, and with epithelial mesenchymal transition: VIM, TGFβ1, ZEB2, FOXC1, CXCR4 [103]. More recently, Lang et al. published a study of RNA expression profiling of breast cancer CTCs using cDNA microarrays [104].

Another source of tumour cell heterogeneity is related to epigenetics, including histone modification, and DNA base modifications (such as methylation and hydroxymethylation), as mentioned previously. Robust technologies have been developed to provide genome-wide maps of most epigenetic marks. ChIP-seq, suitable for studying histone marks at the single-cell level, combines chromatin precipitation with next generation sequencing [104]. To assess methylation levels, the most comprehensive picture can be obtained from whole-genome bisulphite sequencing (WGBS) [89], but precipitation techniques (methylated-DNA immunoprecipitation sequencing and methylated–DNA-binding domain sequencing) or reduced-representation bisulphite sequencing (RRBS) [90] are also used. Illumina’s Infinium Human Methylation450 BeadChip platform provides an array-like alternative that has been found to provide acceptable DNA methylation profiles, reducing the DNA-damaging effects of bisulphite treatment [91].

#### 6.2.2. Single-Cell Proteomic Analysis

Fluorescent Activated Cell Sorting (FACS) data allows disrupted expression patterns in proteins on cells in cancer to be identified, as well as to analyse signalling networks (phosphorylation) and their link to the outcome of the disease. The instruments grouped under the term “flow cytometer” now extend to microfluidics, and miniaturise the components into lab-on-a-chip devices [92,93]. These instruments set a high standard for very quantitative and high-throughput multiparameter measurements of single-cell attributes [105]. Several studies using single-cell phosphorylation analysis by means of flow cytometry, known as phospho-flow, have been performed in cancer, leading to classification of patients into several groups [106], and predicting outcome [107] and drug sensitivity [108].

One of the drawbacks of the flow cytometry analysis is that some pathways may be sensitive to disruption when cells are taken out of context, leading to changes in the intracellular signalling, and this may thus alter results of cell heterogeneity analysis. In whole blood–based assays (i.e., isolating CTCs), this tends to be minimised. However, despite proteomic analyses at the single cell level which have been already successful in haematological malignancies [106,107,108], this approach is just at the beginning for solid tumours [105] and require at least 1000–2800 cells.

Studying proteomics at single-cell level is also possible with Cytometry time of flight (CyTOF), developed by Bandura et al. [109]. CyTOF was developed into an imaging tool that can image the localisation of up to 32 proteins at present (and potentially up to 100) with subcellular resolution [110]. Briefly, this method is based on tagging antibodies with isotopes not found in cells, staining the cells with those tagged antibodies, and then ionising the sample before passing it into a time-of-flight (TOF) mass spectrometer. Ions have a specific mass range and are quantified with the CyTOF instrument. All isotope signals are integrated for each cell and digitised to create a spreadsheet of cell-by-cell information similar to a flow cytometry data file, but containing much more information. This complexity is reduced thanks to analytical approaches such as SPADE [111]. A study performed by Han et al. validated the feasibility of this method for studying single-cell heterogeneity in cancer [112].

## 7. Conclusions and Future Direction

Cancer is a heterogeneous disease, with variations in terms of morphology, immunophenotype and genotype. Analysing conventional genetic profiling on bulk single biopsy tissues is often used today as the standard for evaluating tumours, thus limiting diagnostic and treatment decisions to the analysed region. Profiling of bulk samples may also unravel the phenotypes obtained from previous selective pressures which are not relevant to the status of the current metastatic disease. Furthermore, different regions of the same tumour can contain signatures for either good or bad prognoses; a single biopsy may thus not be sufficiently representative of the tumour’s composition.

Circulating tumour cells (CTCs) can be isolated and interrogated as a potential window into the genetics of a tumour by means of non-invasive sampling, making it possible to evaluate the tumour’s temporal heterogeneity during the clinical course of the disease. To overcome the averaging approach determined by using bulk CTC analysis, single-cell isolation can only be achieved with sophisticated instrumentation, requiring expert operators such as the DEPArray™ system, (Silicon Biosystems, Bologna, Italy) and time-consuming protocols which can only guarantee the single-cell level on rare occasions. Moreover, depending on the methodology used for their isolation, the number of recovered CTCs may be different and thus the analysis of heterogeneity will report different results [5,113]. CTCs have been used successfully to reveal tumour heterogeneity, and can readily be subjected to dynamic monitoring [114]. However, CTCs are present at very low numbers in peripheral blood, and current studies of CTCs are generally limited to advanced cancer. Not all patients with advanced cancer have detectable CTCs, only a few studies have detected CTCs in patients with early-stage cancer [115]. A considerable proportion of cancer patients diagnosed in the early stages develop distant metastases after surgical removal of the primary tumour, indicating that CTCs are present even during the early stages of cancer. A detection system with analytical sensitivity and capable of demonstrating adequate cell recovery and sampling accuracy is needed to successfully isolate and enrich heterogeneous CTCs.

One of the key questions is to determine to what extent CTCs represent heterogeneity within the primary tumour or metastases. It is possible that the cells detected with the currently available techniques are merely those that were shed and are only the “tip of the iceberg”. For example, it is clear that a high number of CTCs detected are associated with poor prognosis, but it is not possible to know if these cells actually have malignant behaviour themselves. Perhaps they are just terminally differentiated cells that merely reflect the presence of more malignant, but uncaptured cancer cells. These cells do not necessarily represent the biology of the underlying tumour [116]. Interestingly, recent data suggest that CTC clusters are able to extravasate through narrow vessels and invade surrounding tissues [53]. In this context, cluster CTCs may have greater metastatic potential than individual CTCs, and identifying and isolating these clusters is a key challenge for the future. Further investigation is needed to both validate this recent discovery and develop new technologies for in-depth study of CTC clusters heterogeneity [53].

In the last ten years, many new technologies have been developed in order to characterise single-cell heterogeneity at the DNA, RNA or protein level. Some of these technologies are reviewed in Section 6. The methodologies present several disadvantages, such as reduced sensitivity for low expressed genes, small quantities of sample material, amplification biases, technical noise or low throughput, among others. In addition, data mined from single-cell studies must be carefully studied with rigorous computational analyses, which are imperative for distinguishing pre-existing genetic alterations from amplification errors. Improper computation may result in bias and errors in data interpretation, especially in highly heterogeneous samples. Independent analyses from multiple single cells may also be used to reveal repeated and specific mutational patterns, which will be pivotal in distinguishing technical noise from biological signals [87]. A complete picture of a cell’s state will often require measurements of different parameters in the same cell (i.e., transcriptome, genome and epigenome). Although it is usually possible to perform multiple assays on a bulk sample, this is only possible in certain cases for single-cell measurements [117]. Further development of multi-model measurement methods will help understand different sources of heterogeneity in a single cell. A major challenge is new multiplexing strategies that can be used to profile single cells quickly and at a reasonable cost. Despite analysis of heterogeneity at the single cell level, it is theoretically a better approach for obtaining the maximum information on tumour heterogeneity. The cost and time required do not allow to use such techniques in routine yet (in clinical practice). The most realistic methods are currently based on analysis of small cell pools. This fact challenges the reliability of overall tumour heterogeneity when it is inferred at the single cell level.

It is important to note that the tumour microenvironment itself is also heterogeneous. This microenvironment, which defines an additional barrier or an asset for therapy, is responsible for differently directed selective pressure within the same tumour, changing the evolutional trajectories of the tumour cells [118]. This opens up new areas of research for developing techniques focusing on regulating the tumour microenvironment in order to control tumour progression. One long-term goal is to move towards tagless, or almost tagless measurements, in which viable cells are isolated prospectively based on function, gene expression and genotypes and then assayed directly for their tumour-initiating capacity. Techniques that probe the three-dimensional composition (architecture and cell types) of tumours, while minimally disrupting cell biology, are also required to gain more information on cell-type location and its relationship to disease. Currently, the only approaches that are readily available (e.g., green fluorescent proteins or other gene expression–based reporters) require genetic modification of the target cell.

## Figures and Tables

**Table 1:** Methodologies for studying the tumour heterogeneity.

Methods	Applications	Main Advantages and Drawbacks	References
Immunohistochemistry (IHC) and Immunofluorescence (IF)	Protein	- Preserved tissue context - Difficult to quantify and to compare between samples - Limitation in the number of analysed markers	[69,70]
Fluorescence in situ Hybridization (FISH)	DNA or RNA		
Immuno-FISH	Genome imbalances and DNA translocations + antigenic markers	- High sensitivity and specificity - Reproducibility - Can be easily automated - Dependent on available probes - No available for high throughput - Only for the analysis of low cell density tumours	[71]
Comparative Genomic Hybridization Array (a-CGH)	DNA copy number variations	- High resolution - Analysis of whole genome - High specificity and sensitivity - Fast technique - Detects only, the copy number changes - No detection mosaicism	[72,73]
RNAscope	RNA	- Compatible with clinical routine practice - Multiple RNA probes can be used at the same time - High sensitivity and specificity - High time-consuming - Complex procedures	[74]
Fluorescent in situ Sequencing (FISSEQ)	mRNA	- Allow the detection of RNA splicing and post-transcriptional modifications (with preservation of their spatial context) - Discrimination of RNA with a small number of reads - Expensive equipment for analysing the results	[75]
Specific-To-Allele PCR–FISH (STARFISH)	Single nucleotide and DNA copy number alterations	- Relative moderated cost - Easy interpretation - Recommended for suspected mutations - Limited number of fluorochromes - Tissue handling affects mRNA expression	[76]
Matrix assisted laser desorption/ionization-imaging mass spectrometry (MALDI-IMS)	Proteins, lipids, metabolites	- Low amount of material can be analysed - Keep the spatial localization information - High sensitivity and molecular specificity - Require accurate sample - Difficulty to control the methods of preparation	[77]
Whole Genome Sequencing (WGS)	DNA: single nucleotide variants, copy number variants, non-coding and structural variants	- Single-base resolution - Deliver large volumes of data in a short amount of time - Suitable for discovering of new markers - Require high skills for data handling and interpretation - Relatively high cost	[78,79,80,81,82,83,84]
Whole Transcriptome Sequencing (WTS)	mRNA	- High throughput analyses - Single-base resolution - Low amount of sample required - Sample handling must be accurate - Complex procedures	[85,86]
Multiplexed error-robust FISH (MERFISH)	RNA	- Conservation of the cell spatial information - High throughput analyses - Predesigned probes (limited discovery capacity)	[87]
Chromatin ImmunoPrecipitation Sequencing (ChIP-Seq)	DNA/protein binding, histone marks	- Single nucleotide resolution - Repetitive regions in the genome can be analysed - Require large amounts of starting material - Low sensitivity and high technical read variance - Relatively high cost	[88]
Whole Genome Bisulfite Sequencing (WGBS)	Methylation of whole genome	- Low quantity of starting material - Relatively high cost	[89]
Reduced Representation Bisulfite Sequencing (RRBS)	Methylation of whole genome	- High throughput analyses - Low amounts of starting material - Multiple step procedure (Risk: accumulation of errors)	[90]
Flow Cytometry into lab-on-a-chip	Protein	- Quantitative technic - High throughput analyses - Multiparameter measurements - Some pathways can be disrupted when cells are taken out of context	[91,92,93]
Mass Cytometry (CyTOF)	Protein	- No spectral overlap of detectors - Up to 32 proteins can be detected simultaneously - Slow acquisition - Limited commercially labelled antibodies - Complex data to analyse	[91,92,93]
